# PSMC2/ITGA6 axis plays critical role in the development and progression of hepatocellular carcinoma

**DOI:** 10.1038/s41420-021-00585-y

**Published:** 2021-08-19

**Authors:** Xuhua Duan, Hao Li, Manzhou Wang, Shuguang Ju, Fengyao Li, Pengfei Chen, Huibin Lu, Xinwei Han, Jianzhuang Ren

**Affiliations:** Department of Interventional Radiology, The First Affiliated Hospital, Zhengzhou University, Zhengzhou, Henan China

## Abstract

Hepatocellular carcinoma (HCC) is a type of malignant tumor with sixth highest incidence and causes the third most cancer-related deaths in the world, whose treatment is limited by the unclear molecular mechanism. Currently, the correlation between PSMC2 and HCC is still unclear. Herein, we found that the expression of PSMC2 in HCC tissues was significantly higher than normal tissues. We also discovered the significant association between PSMC2 expression and tumor infiltrate as well as tumor stage. Further investigations indicated that PSMC2 knockdown contributed to impaired proliferation, colony formation, migration, and enhanced cell apoptosis in HCC cells. Moreover, PSMC2 could also suppress tumorigenicity of HCC cells in vivo. Gene microarray analysis followed by ingenuity pathway analysis was performed for exploring downstream of PSMC2 and identified ITGA6 as a potential target. Furthermore, our study revealed that ITGA6 knockdown exhibited similar inhibitory effects with PSMC2 on HCC cells in vitro. More importantly, our results proved the direct interaction and showed the mutual regulation between PSMC2 and ITGA6, and that PSMC2 knockdown could significantly aggravate the inhibition of HCC by ITGA6 depletion. Based on these intriguing results, this is the first time ever that PSMC2 is pinpointed as a tumor promotor to interfere HCC development and progression via interacting with ITGA6 directly.

## Introduction

As the malignant tumor with sixth highest incidence and the third leading cause of cancer-related death in the world, hepatocellular carcinoma (HCC) has been paid more and more attention in clinical and basic medical research [1, 2]. Now, the treatment options for HCC patients include surgical treatment, local ablation, transcatheter arterial chemoembolization, radiotherapy, and systemic therapy [3–5]. However, due to the occult and rapid development of HCC, most patients have progressed to intermediate and advanced stage when diagnosed, which seriously disturbed the effects of HCC treatment, ultimately leading to a shorter survival rate and poor prognosis of HCC patients [6]. In the past few decades, the concept of molecular targeted drug brought an exciting and evolutionary progress to cancer treatment [7]. However, because of the insufficient understanding of the molecular mechanism of HCC, only two molecular targeted drugs (Sorafenib and Lenvatinib) have been approved by FDA as first-line therapy for HCC patients, whose therapeutic efficiency was seriously limited by the decrease of drug tolerance and the emergence of drug resistance in patients with HCC [8, 9]. Although a variety of HCC targeting drugs are in clinical trials and show some advantages, it is of significant urgency to deepen the understanding of molecular mechanism of HCC and identify more effective targets [10].

The ubiquitin/26S proteasome pathway is an efficient protein degradation pathway, mainly responsible for the selective degradation of proteins in eukaryotic cells [11]. As far as current knowledge is concerned, the 26S proteasome is a multi-catalytic enzyme complex, which is abundantly expressed in the nucleus and cytoplasm of all eukaryotic cells [12]. In addition, one of the outstanding functions of the 26S proteasome is to participate in the regulation of the expression levels of various proteins that play a central role in cell processes, such as cell cycle progress, signal transcription, DNA repair, metabolic regulation, and cell apoptosis [13]. Altered regulation of these cellular events is linked to the development and progression of cancer. Therefore, much work has focused on the proteasome as a promising target for the treatment of numerous cancers [14]. Proteasome 26S subunit ATPase 2 (PSMC2) located in 7q22.1-q22.3 of the genome is an indispensable member of the 19S regulatory subunit of 26S proteasome and engaged in catalyzing substrates and transporting them into the 20S proteasome [15]. Reviewing previous research, the author demonstrated that PSMC2 knockdown in tumor cells could inhibit tumor development and that PSMC2 was identified as a potential gene related to certain human tumors. In addition, He et al. reported that the higher PSMC2 expression was associated with poorer survival rate of colorectal cancer patients and that PSMC2 silencing in colorectal cancer cells suppressed cell proliferation and migration, and induced cell apoptosis [16]. Despite that PSMC2 is considered to be a newly discovered gene closely related to human cancer, its expressional characteristics and functional value in HCC is pending to be explored.

In this study, immunohistochemical staining was performed on clinical specimens to show the differential expression of PSMC2 in HCC and normal tissues. The statistical analysis of the association between PSMC2 expression and tumor characteristics of HCC patients was also employed to clarify the effects of PSMC2 on HCC development and progression. Lentivirus expressing PSMC2-targeting shRNA was used for silencing PSMC2 in HCC cells, thus investigating its regulatory ability in cell proliferation, colony formation, and cell apoptosis in vitro or in vivo. Moreover, the potential mechanism for PSMC2 to promote HCC was further explored by RNA sequencing and verified by rescue experiments. To the best of our knowledge, this study is the first attempt to illustrate the potential oncogenic activity of PSMC2 in HCC.

## Results

### PSMC2 were upregulated in HCC tissues and expressed in HCC cells

**Fig. 1:**
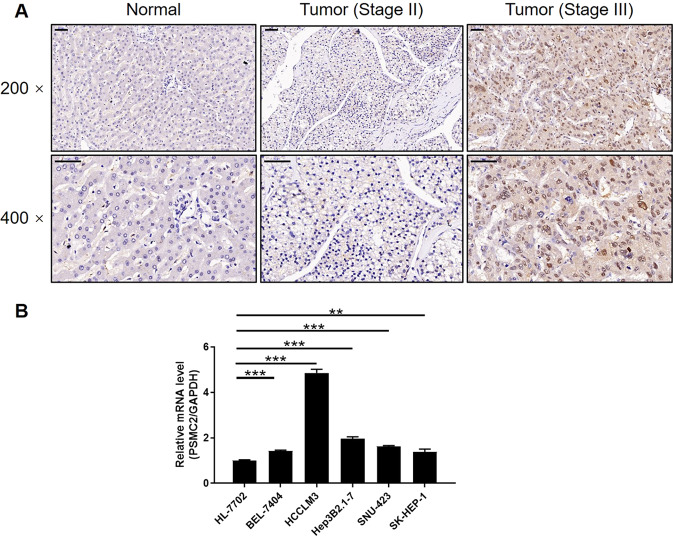
PSMC2 was upregulated in HCC tissues and expressed in HCC cells. **A** The PSMC2 expression was evaluated by IHC analysis in HCC tissues and normal tissues (scale bar = 50 μm). **B** The mRNA expression of PSMC2 in normal cell line HL-7702 and HCC cell lines including HCCLM3, SK-HEP-1, BEL-7404, SNU423, and Hep3B2.1-7 was determined by qPCR. The representative images were selected from at least three independent experiments. Data were shown as mean ± SD. **P < 0.01, ***P < 0.001.

Immunohistochemistry (IHC) analysis was used to evaluate the role of PSMC2 in HCC and to visualize the role of PSMC2 expression in HCC and normal tissues. The expression levels of PSMC2 were significantly upregulated in HCC tissues (Fig. 1A) compared with normal ones. The significant elevation in PSMC2 expression levels in HCC was also demonstrated by quantifying its expression in 108 and 10 HCC and normal tissues, respectively (*P* < 0.001, Table 1). We also identified significantly higher expression levels of PSMC2 in HCC patients with higher levels of T infiltrate and more advanced tumor stages (Fig. 1A). This was also verified by correlating the PSMC2 expression levels and the clinical characteristics of tumors in HCC patients (*P* < 0.05, Table 2) and by Pearson correlation analysis (Supplementary Table S3). The higher expression levels of PSMC2 in more serious cases of HCC suggested that this protein plays a key role in the development of HCC. With the exception of HCC tissues, qPCR detection also revealed the generally higher levels of PSMC2 in a number of HCC cell lines, including HCCLM3, SK-HEP-1, BEL-7404, SNU423, and Hep3B2.1–7 than normal cell line HL-7702 (Fig. 1B). Of these, based on our preliminary experiments that are not shown in this study, we selected SK-HEP-1 and BEL-7404 cell lines for subsequent investigations.

**Table 1:** Expression patterns of PSMC2 in HCC tissues and normal tissues revealed in immunohistochemistry analysis.

PSMC2 expression	Tumor tissue		Normal tissue	
	Cases	Percentage	Cases	Percentage
Low	50	46.3	10	100
High	58	53.7	0	–

*P* < 0.001.

**Table 2:** Relationship between PSMC2 expression and tumor characteristics in patients with HCC.

Features	No. of patients	PSMC2 expression		*P* value
		Low	High	
*All patients*	108	50	58	
*Age (years)*				0.117
<51	52	20	32	
≥51	56	30	26	
*Gender*				0.024
Male	84	34	50	
Female	24	16	8	
*Grade*				0.198
I	15	4	11	
II	73	36	37	
III	14	7	7	
*Stage*				0.031
I	6	5	1	
II	46	24	22	
III	56	21	35	
*T Infiltrate*				0.012
T1	6	5	1	
T2	48	26	22	
T3	48	17	31	
T4	6	2	4	

### The in vitro development of HCC was inhibited by the depletion of PSMC2

**Fig. 2:**
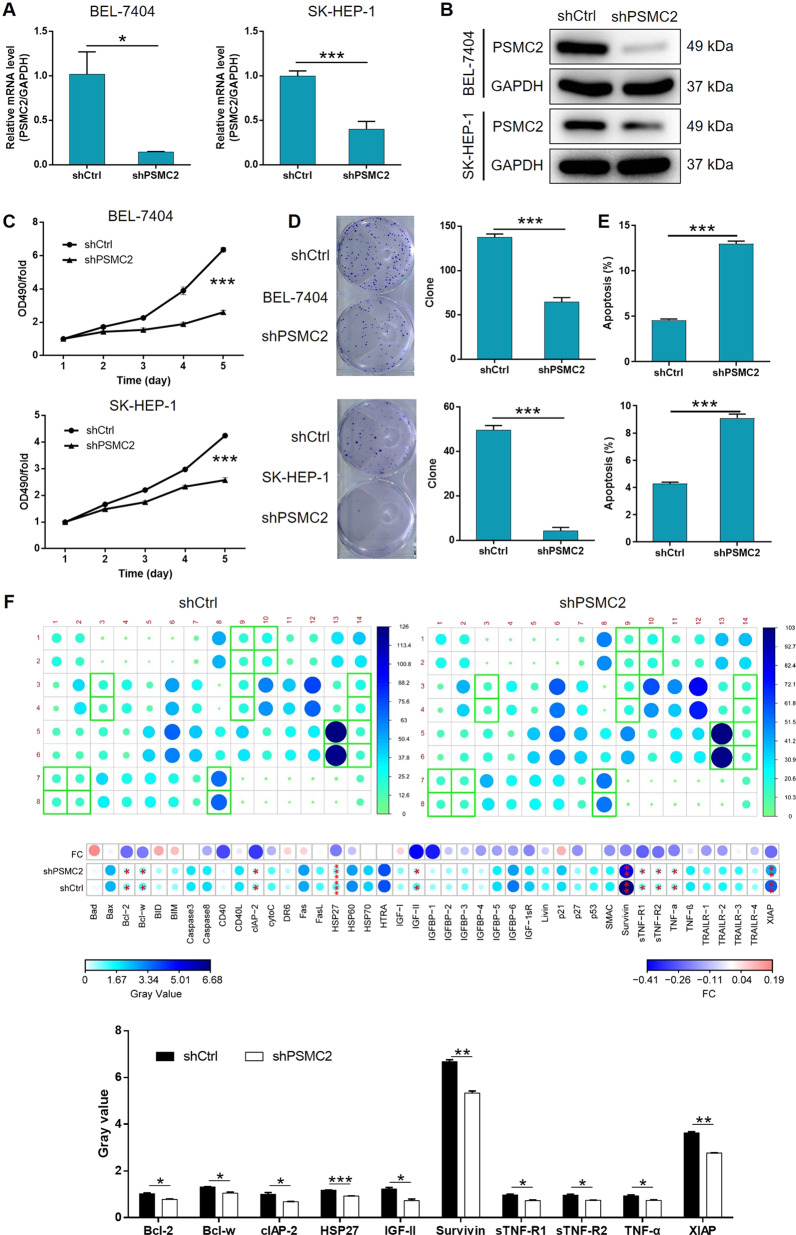
PSMC2 knockdown inhibited HCC development in vitro. **A**, **B** Cells transfected with shPSMC2 or shCtrl were employed to build the cell model. The knockdown efficiency of PSMC2 in BEL-7404 and SK-HEP-1 cells was estimated by qPCR (**A**) and western blotting (**B**). Herein, the GAPDH blot was shared with Supplementary Figs S2 and S5C. **C** MTT assay was utilized to indicate proliferation ability of BEL-7404 and SK-HEP-1 cells influenced by PSMC2. **D** Plate clone formation assay was applied to investigate the colony formation ability of BEL-7404 and SK-HEP-1 cells with or without knocking down PSMC2. **E** Flow cytometry was performed to detect the effects of PSMC2 on cell apoptosis of BEL-7404 and SK-HEP-1 cells. **F** The role of PSMC2 in apoptosis-related protein expression in SK-HEP-1 cells was analyzed by Human Apoptosis Antibody Array. Data were shown as mean ± SD. *P < 0.05, **P < 0.01, ***P < 0.001.

Next, we transfected HCC cells with lentivirus to knockdown PSMC2 and investigate the effects of its absence in HCC. We observed fluorescence signals in >80% of cells, thus demonstrating that the transfection had been successful (Supplementary Fig. S1A). We selected the shRNA with the best efficiency to knockdown PSMC2 by qPCR screening and used this shRNA for subsequent experiments (Supplementary Fig. S1B). qPCR and western blotting revealed the significant downregulation of PSMC2 at the mRNA and protein levels (Fig. 2A, B), respectively. This confirmed the successful knockdown of PSMC2 in both SK-HEP-1 and BEL-7404 cell lines. MTT assays clearly demonstrated that HCC cells in which PSMC2 had been knocked down (shPSMC2) exhibited significantly slower proliferation rates than those with relatively higher levels of PSMC2 expression (shCtrl) (*P* < 0.001, Fig. 2C). The ability of HCC cells to successfully form colonies was also seriously reduced following the knockdown of PSMC2 knockdown (*P* < 0.001, Fig. 2D). Next, we investigated the extent of cell apoptosis in HCC cells with or without PSMC2 depletion by flow cytometry. We also found that the proliferation of HCC cells was inhibited since more apoptotic cells were detected in the shPSMC2 group compared to the shCtrl group (*P* < 0.001, Fig. 2E). We also carried out a human antibody array analysis in order to identify differential protein expression levels associated with cell apoptosis that might be mediated by PSMC2 and attempted to identify the regulatory mechanisms involved. The absence of PSMC2 absence led to the impaired expression of several proteins, including containing Bcl-2, Bcl-w, clAP-2, HSP27, IGF-II, Survivin, sTNF-R1, sTNF-R2, TNF-α, and XIAP (Fig. 2F). Moreover, the elevated expression of Caspase-3 and Caspase-7 in shPSMC2 cells also proved the promoted cell apoptosis by PSMC2 knockdown (Supplementary Fig. S2). Notably, we also validated the suppression of cell growth, and the enhancement of cell apoptosis, in cells in which PSMC2 had been knocked down by carrying out additional experiments in a third cell line, HCCLM3 (Supplementary Fig. S3). Our experiments suggested that PSMC2 may play a critical role in the development of HCC development by exerting effects on cell apoptosis, colony formation, and apoptosis.

### HCC may be regulated by PSMC2 by targeting ITGA6

**Fig. 3:**
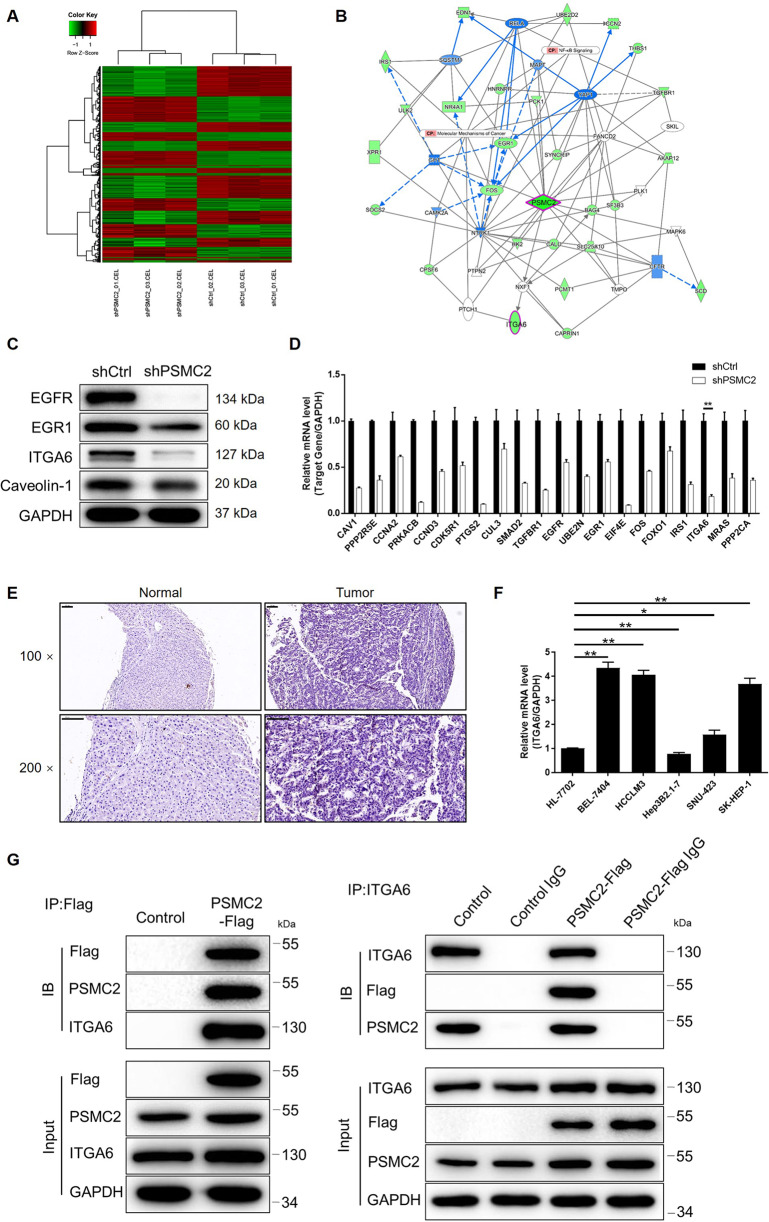
The exploration and verification of downstream underlying PSMC2-mediated regulation in HCC. **A** A PrimeView Human Gene Expression Array was conducted to distinguish the differentially expressed genes (DEGs) of SK-HEP-1 cells with or without silencing PSMC2. **B** A PSMC2-induced interaction network was established based on IPA analysis. Line: interaction; arrow: regulation; full line: direct regulation; dotted line: indirect regulation; green: downregulation; blue: predicted inhibition; white: with state of downstream molecule. The expression of several selected DEGs in SK-HEP-1 cells with or without PSMC2 knockdown was detected via qPCR (**C**) and western blotting (**D**). **E** The ITGA6 expression in HCC tissues and normal tissues was evaluated by IHC analysis (scale bar = 100 μm). **F** The mRNA expression of PSMC2 in normal cell line HL-7702 and HCC cell lines including HCCLM3, SK-HEP-1, BEL-7404, SNU423, and Hep3B2.1-7 was detected by qPCR. **G** A co-IP assay was performed to show the direct interaction between PSMC2 and ITGA6. The representative images were selected from at least three independent experiments. Data were shown as mean ± SD. *P < 0.05, **P < 0.01.

Our previous results indicated that PSMC2 was involved in the development of HCC. Next, we attempted to investigate the potential mechanisms underlying this effect. We used 3 v 3 (shCtrl vs shPSMC2) gene microarray analysis to distinguish differentially expressed genes (DEGs) between SK-HEP-1 cells with or without PSMC2 deficiency. Using specific thresholds (|fold change| ≥ 2.0 and FDR < 0.05 (*P* values obtained via Benjamini–Hochberg analysis), we identified 621 upregulated DEGs cells from the shPSMC2 group when compared to cells from the shCtrl group; we also identified 709 downregulated DEGs in cells from the shPSMC2 group (Supplementary Fig. S4A and Fig. 3A). Ingenuity pathway analysis (IPA) was performed to show the enrichment of the 1330 DEGs in the canonical signaling pathway as well as IPA disease and function. Notably, the protein ubiquitination pathway was the most enriched signaling pathway; cancer was the most enriched candidate with regards to IPA disease and function (Supplementary Fig. S4B, C). On the basis of our bioinformatics analysis and the IPA analysis, we created a PSMC2-related interaction network (Fig. 3B) and several DEGs were selected for validation by qPCR and western blotting (Fig. 3C, D). Of these, ITGA6, a member of one of the most enriched CDK5 signaling pathways, was also significantly downregulated in SK-HEP-1 cells in the absence of PSMC2; this suggested that ITGA6 may represent a potential target for PSMC2 (Supplementary Fig. S4D and Fig. 3B, D). Indeed, in a manner that was similar to PSMC2, ITGA6 was significantly upregulated in HCC tissues and HCC cell lines when compared to normal tissues and normal cell line, respectively (Fig. 3E, F). Data collected from TCGA database also indicated a positive correlation between PSMC2 and ITGA6 in HCC tissues (Supplementary Fig. S4E). Furthermore, we found the upregulation of ITGA6 in HCC tissues in comparison with normal tissues (GSE121248 dataset of GEO database), and the significant correlation between high ITGA6 expression and poor prognosis of HCC patients (TCGA database) (Supplementary Fig. S5A, B). More importantly, co-IP results provided evidence to support the fact that ITGA6 can interact directly with PSMC2; ITGA6 was detected in a complex precipitated by anti-PSMC2-Flag and vice versa (Fig. 3G). Further western blotting indicated the downregulation of ITGA6 in shPSMC2 HCC cells (Supplementary Fig. S5C). Collectively, our data showed that ITGA6 represents a promising target that might play a role in regulating the development of HCC via PSMC2.

### The knockdown of PSMC2 aggravated the inhibition of HCC by depleting ITGA6

**Fig. 4:**
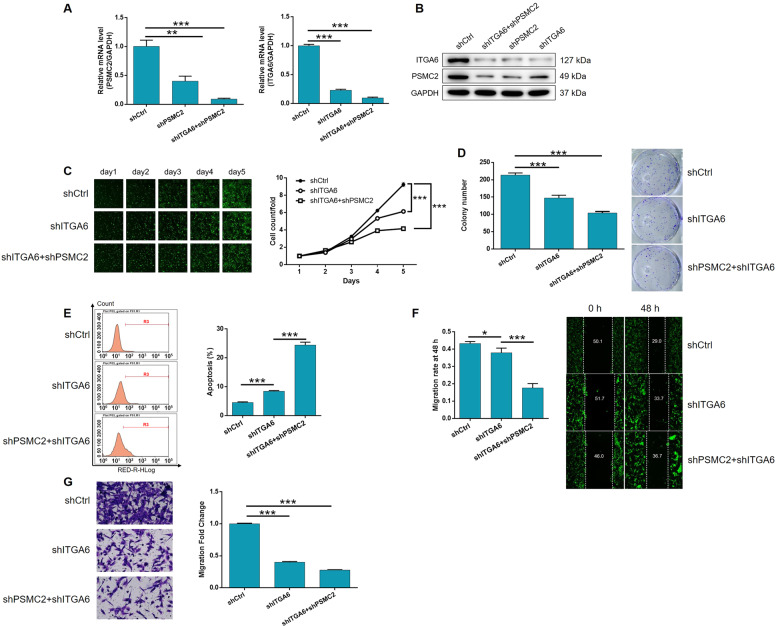
Knockdown of PSMC2 deepens the effects on HCC cells by ITGA6 knockdown. **A**, **B** The expression of PSMC2 and ITGA6 in SK-HEP-1 cells transfected with shCtrl, shITGA6 and simultaneous shPSMC2 and shITGA6 were detected by qPCR (**A**) and western blotting (**B**). Cell models were adopted in the detection of cell proliferation by Celigo cell counting assay (**C**), colony formation (**D**), cell apoptosis (**E**), and cell migration by wound-healing assay (**F**) and Transwell assay (**G**). The representative images were selected from at least three independent experiments. Data were shown as mean ± SD. *P < 0.05, **P < 0.01, ***P < 0.001.

Next, we established a SK-HEP-1 cell model transfected with shITGA6, or both shPSMC2 and shITGA6, in order to investigate their combined effects on HCC. First, we evaluated transfection efficiency using a method that was described previously and then used qPCR to identify the most effective shRNA for silencing ITGA6 (Supplementary Fig. S6). Figure 4A, B demonstrates the mutual regulation between PSMC2 and ITGA6: (1) the depletion of PSMC2 downregulated ITGA6 while ITGA6 deficiency led to the downregulation of PSMC2; (2) PSMC2 was downregulated in the shITGA6 group but showed greater levels of downregulation in the shPSMC2+shITGA6 group; (3) the severity of ITGA6 downregulation was higher in the shPSMC2+shITGA6 group than in the shITGA6 group (Fig. 4A, B). Subsequent analysis revealed the strong inhibitory effects of ITGA6 knockdown on cell proliferation and colony formation, and clear stimulative effects on cellular apoptosis (*P* < 0.001, Fig. 4C–E and Supplementary Fig. S7); similar effects were evident for PSMC2. Moreover, as shown in Fig. 4F, G, wound-healing and Transwell assays suggested that the depletion of ITGA6 significantly restrained the ability of SK-HEP-1 cells to migrate (*P* < 0.05 for wound-healing assays, *P* < 0.001 for Transwell assays). Furthermore, we demonstrated that the additional knockdown of PSMC2 in cells in which ITGA6 had already been knocked down, could aggravate the effects on all the observed cellular functions, including cell proliferation, colony formation, apoptosis, and migration (*P* < 0.001, Fig. 4C–G and Supplementary Fig. S7). Collectively, these data indicated the role of the PSMC2/ITGA6 axis in HCC development.

### PSMC2 suppressed tumor growth in vivo

**Fig. 5:**
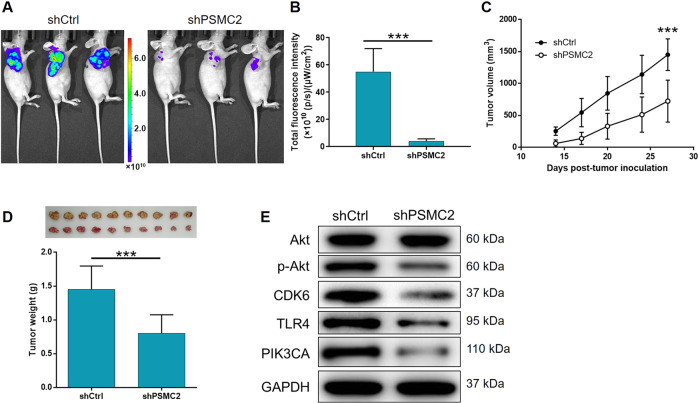
PSMC2 knockdown inhibited HCC development in vivo. **A** In vivo imaging was utilized to estimate the tumor burden in mice of shPSMC2 and shCtrl groups at day 27 post tumor-inoculation. **B** The total fluorescence intensity was analyzed to represent tumor burden in mice of shPSMC2 and shCtrl groups. **C** Tumor volume of mice at 14 days post injection was measured and calculated at indicated time intervals. Inset: photo of the removed tumors was taken at day 27 post tumor-inoculation. **D** Removed tumors were photographed and weighed. **E** The expression of Akt, p-Akt, CDK6, TLR4, and PIK3CA was determined by western blotting in SK-HEP-1 cells with different expression level of PSMC2. Data were shown as mean ± SD. ***P < 0.001.

For in vivo study, we successfully established a mouse xenograft model by injecting HCCLM3 cells transfected with either shPSMC2 or shCtrl. The analysis of in vivo fluorescence imaging indicated that the shPSMC2 group exhibited markedly weaker signals and a lesser extent of tumor burden (*P* < 0.001, Fig. 5A, B). As shown in Fig. 5C, D, both tumor volume and weight were significantly lower in the shPSMC2 group, thus indicating that the absence of PSMC2 led to an inhibition in tumor growth (*P* < 0.001).

### Mechanistic screening

Finally, we used western blotting to detect the expression levels of several well-known key molecules in the cancer-associated signaling pathway so that we could investigate the regulatory mechanisms that link PSMC and /ITGA6 with HCC. As indicated by Fig. 5E, the levels of Akt phosphorylation, and the expression levels of CDK6, TLR4, and PIK3CA, were significantly downregulated by the knockdown of PSMC2, thus indicating the potential involvement of PSMC2 in the regulation of HCC.

## Discussion

To our best knowledge, the 26S proteasome includes the 20S core catalytic subunit and 19S regulatory subunit, which is one of the multimeric ubiquitin-mediated proteasomes [11, 17]. Accumulating studies provided evidence that proteasome inhibitors played a critical role in anti-tumor with the help of the endoplasmic reticulum stress caused by 26S proteasome inhibition [18]. On the other hand, in view of the multiple important roles played by 26S proteasome in the biological processes, the basic research on 26S proteasome has attracted widespread attention, especially in the prospective prevention and treatment of malignant tumors [14]. PSMC2 is ranked as the top among 56 candidate genes in the Copy number alterations Yielding Cancer Liabilities Owing to Partial losS (CYCLOPS) genes, indicating the essential importance of PSMC2 in cancer cell viability [19]. At the same time, considerable research efforts have identified the important role of PSMC2 in some human cancers. For instance, the results from Song et al. described that the upregulated PSMC2 had a promotion effect on the development and progression of osteosarcoma via regulating osteosarcoma cell phenotype including cell proliferation, apoptosis, and migration as well as colony formation [20]. Furthermore, another report further provided a deeper understanding of the relationship between PSMC2 and osteosarcoma based on the result that miR-630 promoted osteosarcoma cell proliferation, migration, and invasion by targeting PSMC2 [21]. Nevertheless, the relationship between PSMC2 and HCC has not been reported and still remains unknown.

This is the first report presented ever to identify PSMC2′s value in HCC development and progression. First, the measurement of PSMC2 expression in clinical specimens established the linkage between high expression of PSMC2 and more serious HCC (more serious T infiltrate and more advanced tumor stage). Through comparing the difference of cell functions of HCC cells with or without PSMC2 knockdown, it was revealed that cells with relatively low expression of PSMC2 exhibited slower cell proliferation rate, weaker ability to form colonies, and were inclined to apoptosis. All the results of in vitro studies suggested the oncogene-like properties of PSMC2 in HCC. Consistently, cells with PSMC2 knockdown showed weaker tumorigenicity and the formed tumors also grew relatively slower in vivo. Moreover, we further found that apoptosis-related proteins consisting of Bcl-2, Bcl-w, clAP-2, HSP27, IGF-II, Survivin, sTNF-R1, sTNF-R2, TNF-α, and XIAP, were significantly downregulated in HCC cells with PSMC2 knockdown, which contributed to the regulation of PSMC2 in cell proliferation and apoptosis,.

The potential downstream of PSMC2 was screened through RNA-seq in this study. As a member in one of the most DEGs enriched signaling pathways, ITGA6 (alias integrin α6) was demonstrated to have direct interaction with PSMC2 and recognized as a potential key factor in the PSMC2-induced regulation of HCC. Integrin is a kind of transmembrane glycoprotein, which can combine with extracellular matrix to regulate the adhesion between cell and matrix or cell and cell. Accumulating evidence has illustrated that integrin expression in different stages of tumor development is also regulated to varying degrees, endowing it the potential as target for cancer diagnosis and treatment [22, 23]. ITGA6 contains 1130 amino acids, which can form α6β1 or α6β4 dimer by binding with β1 or β4 subunits, and can bind with extracellular matrix laminin [24]. At present, there are few research studies on the biological function of ITGA6, among which tumor-related research studies are mainly focused on breast cancer. Cariati et al. found that the abnormal expression of ITGA6 could promote the occurrence and development of breast cancer, which may be a potential target of anti-cancer therapy [25]. Further study reported by Brooks et al. identified ITGA6 as a downstream target of HIF-1α that participated in the HIF-1α-induced stemness and metastasis of breast cancer [26]. Moreover, ITGA6 could mediate radiation resistance of breast cancer through activating Akt/ERK signaling pathway [27]. On the other hand, Laudato et al. demonstrated that ITGA6, together with ITGB1, was target of miR-30e-5p in colorectal cancer [28]. Zhang et al. provided evidence that the tight association existed between ITGA6 overexpression and human gallbladder carcinoma with invasive, metastatic, and poor prognostic features [29]. Despite that the α6β4 dimer was previously revealed to be involved in HCC [30], the expression pattern, biological behavior, and molecular mechanism of ITGA6 in HCC are still unclear. Overexpression of ITGA6 in HCC tissues and cell lines was observed in this study, depletion of which inhibited HCC cell proliferation, colony formation, and migration, and induced cell apoptosis simultaneously. More importantly, PSMC2 deficiency led to the downregulation of ITGA6, and vice versa, indicating the interaction between PSMC2 and ITGA6. Furthermore, simultaneous knockdown of PSMC2 and ITGA6 suppressed cell proliferation and migration, adding to the inhibitory effects of mere ITGA6 knockdown.

It is well known that Akt signaling pathway possesses critical functions in a variety of biological processes and diseases including cancer [31–33]. In our study, it was found that the phosphorylation level of Akt was clearly downregulated in PSMC2 knockdown cells, indicating the activating Akt signaling by PSMC2 in HCC. Toll-like receptor is a type I transmembrane receptor discovered in recent years. Among the 11 TLRs found, TLR4 is expressed in many kinds of tumor cells, which promotes the development of tumors, especially inflammation-related ones through different signaling pathways, and plays an important role in cancer pain and tumor immune escape [34–36]. Herein, the downregulated expression of TLR4 was observed in PSMC2-deficient HCC cells, which was also in agreement with previous reports [37]. Despite of the above findings, it is still unclear and requires further investigations to delineate the mechanism during the regulation of PSMC2 in HCC development and metastasis.

In summary, PSMC2 was found to be an important regulator in the development and progression of HCC. The expression of PSMC2 is relatively higher in HCC tissues than normal tissues, which was also related to the severity of HCC. In vitro and in vivo studies verified that HCC development could be restrained when endogenous PSMC2 was knocked down. Moreover, we also revealed that PSMC2 may execute its regulatory effects on HCC through interacting ITGA6. Altogether, PSMC2 may be a potential therapeutic target of HCC, whose regulatory mechanism needs to be elucidated in the future.

## Material and methods

### Clinical tissue sections and cell culture

HCC tissue and normal tissue sections were obtained from Xi’an Alenabio. Co., Ltd (Shanxi, China). HCC cell lines (SK-HEP-1, BEL-7404, HCCLM3, Hep3B2.1-7, and SNU423) were purchased from BeNa Technology (Hangzhou, Zhejiang, China) and cultured in 90% RPMI-1640 (# 31800022, GIBCO, Rockville, MD, USA) supplemented with 10% fetal bovine serum (FBS) (#10099-141, GIBCO, Rockville, MD, USA) and were cultured at 37 °C in a humidified incubator with 5% CO_2_, and maintained fresh medium every 3 days.

### Immunohistochemistry (IHC)

HCC and normal tissue sections were used for IHC analysis to evaluate PSMC2 expression in different tissues. In brief, after baked at 60 °C for 1 h, the chip was dehydrated by xylene and ethanol solutions (100%, 95%, 90%, and 70%). Then antigen retrieval was conducted using citric acid buffer. Next, the chip was blocked, and added with the primary antibody for incubation at 4 °C overnight. After that, washed by PBS, the chip continued to be incubated with the second antibody for 2 h at room temperature (information of antibodies shown in Supplementary Table S1). The last procedure was performed to stain the chip with DAB and hematoxylin, photograph, and analyze it via ImageScope and CaseViewer software. IHC scores were calculated based on staining percentage and staining degree. Typically, staining percentage scores were judged as 1 (1–24%), 2 (25–49%), 3 (50–74%), 4 (75–100%). Staining intensity were scored as 0 (signalless color), 1 (brown), 2 (light yellow), 3 (dark brown). This study was approved by Ethical Committee of the First Affiliated Hospital, Zhengzhou University.

### Construction and package of lentiviral victors

Short hairpin RNAs for PSMC2 (target sequence: 5′-GCCAGGGAGATTGGATAGAAA-3′) and ITGA6 (target sequences: 5′-AACCAGCAAGGCAGATGGAAT-3′; 5′-AACCATCACAGTAACTCCTAA-3′; 5′-GAGCCCAAATATACTCAAGAA-3′) were designed by Shanghai Biosciences, Co., Ltd (Shanghai, China), respectively. These RNAs were further cloned into the BR-V-108 plasmid using digestion of the restriction enzymes Age I (#R3552L) and EcoRI (#R3101L) (NEB, Beijing, China) to construct complete plasmids. Sequences in the recombinant plasmids were verified with PCR. Plasmids were extracted using EndoFree Maxi Plasmid Kit (#Y5-12381, Qiagen, Netherlands, Hilden, Germany), the quality of which was assessed via Thermo Nanodrop 2000 (Waltham, MA, USA) under the guidance of manufacturer’s manuals. Qualified plasmid was used for packaging.

### Cell transfection

HCC cell lines (SK-HEP-1, HCCLM3, and BEL-7404) were incubated with lentiviral vectors (4 × 10^8^ TU/mL), ENI.S and Polybrene (Sigma-Aldrich, St Louis, MO, USA) in 6-well plates. After cultured at 37 °C with a 5% CO_2_ for 72 h, the fluorescence was observed under microscope with magnification of ×100 and ×200.

### qRT-PCR

TRIzol reagent (Sigma, St. Louis, MO, USA) was used to extract RNA from cells. Nanodrop 2000/2000C spectrophotometer (Thermo Fisher Scientific, Waltham, MA, USA) was used to estimate RNA quality according to the manufacturer’s instructions. Then cDNA was reversely transcribed from RNA using Promega M-MLV kit (Heidelberg, Germany). Afterwards, SYBR Green mastermixs Kit (Vazyme, Nangjing, Jiangsu, China) was employed in qRT-PCR analysis. At last, 2^−ΔΔCt^ method was referred to quantitate the gene expression level. GAPDH was set as an internal control. All primer sequences of human PSMC2 during this assay are displayed in Supplementary Table S2.

### Western blotting and co-immunoprecipitation

Total protein was collected after lysing cells in ice-cold RIPA buffer (Millipore, Temecula, CA, USA), and the protein concentration was determined by BCA Protein Assay Kit (#23225, Pierce, Logan, UT, USA). Protein (20 μg per lane) was separated by 10% SDS-PAGE (Invitrogen, Carlsbad, CA, USA) and then was transferred onto PVDF membranes at 4 °C. After blocking with 5% degreased milk TBST solution, these membranes were incubated at 4 °C overnight with related primary antibodies, washed by TBST, and added with second antibody for 2 h at room temperature subsequently. After the visualization of proteins using ECL-PLUS Kit (#RPN2232, Amersham, Chicago, IL, USA), the target protein density was evaluated via software. Antibodies used in WB assay are shown in Supplementary Table S1.

Before performing co-immunoprecipitation, SK-HEP-1 cells were overexpressed with Flag-labeled PSMC2. Immunocomplexes were collected from whole cell lysate through the immunoprecipitation by anti-Flag or anti-ITGA6 antibody, followed by the western blotting analysis by both anti-Flag and anti-ITGA6 antibodies.

### MTT assay

Lentivirus transfected SK-HEP-1, BEL-7404, and HCCLM3 cells were seeded into a 96-well plate at a density of 2000 cells/well. OD490 were detected 24, 48, 72, 96, and 120 h post cell seeding. Four hour before each detection point, living cells were colored with 20 μL MTT solution (5 mg/mL, # JT343, GenView, El Monte, CA, USA). and DMSO (#130701, Shanghai Shiyi, China) was the solvent of formazan. OD490 were detected via a microplate reader (Tecan, Männedorf, Zürich, Switzerland).

### Cell apoptosis assay

Lentivirus transfected SK-HEP-1, BEL-7404, and HCCLM3 cells were inoculated in a 6-well plate. Cells were collected in binding buffer, and incubated with Annexin V-APC (eBioscience, San Diego, CA, USA) at room temperature for 15 min in dark. Finally, FACSCalibur (BD Biosciences, San Jose, CA, USA) was used to analyze apoptotic cells.

### Colony formation assay

Target sequence lentivirus transfected SK-HEP-1 and BEL-7404 cells were collected 5 days post transfection. Each experimental group cells were digested with trypsin (#T0458-50G, Shenggong, Shanghai, China), resuspended to be seeded in a 6-well plate (350 cells/mL) and further cultured for 8 days. After fixed using 4% paraformaldehyde (SIGMA, St Louis, MO, USA) and stained with GIEMSA (Shanghai Dingguo, China), all colonies were photographed with a digital camera and colony number was counted.

### Celigo cell counting assay

Seventy-two hours after the transfection, SK-HEP-1 cells were collected and seeded into a 96-well plate with 3000 cells per well. Cells were further cultured in RPMI-1640 medium containing 10% FBS. Cells were pictured and counted automatically using Celigo image cytometer (Nexcelom Bioscience, Lawrence, MA, USA) at 1, 2, 3, 4, and 5 days post tranfection. According to the data collected, cell proliferation curve was drawn and fold change value was calculated.

### Wound-healing assay

LV-shCtrl, LV-shITGA6, and LV-(shITGA6+shPSMC2) transfected SK-HEP-1 cells were seeded into a 96-well dish at a density of 4 × 10^4^ cells per well with three replicates. Twenty-four hours later, the medium was replaced with low concentration serum medium, and a 96-wounding replicator (VP scientific, San Diego, CA, USA) was employed to make line wounds. Cell monolayers were washed with serum-free medium, and added with 0.5% FBS medium. Cell images were captured via fluorescence microscope at 0 and 48 h after making wounds. Migration area was calculated using ArrayScan VT1 Cellomics (Thermo Fisher Scientific, Waltham, MA, USA).

### Transwell assay

LV-shCtrl, LV-shITGA6, and LV-(shITGA6 + shPSMC2) transfected SK-HEP-1 cells were resuspended with serum-free medium and seeded into upper chamber of a 24-well Transwell migration insert (#3422, Corning, NY, USA) with 5 × 10^5^ cell/well, and three wells for each group with 100 μL serum-free medium per well. The lower chamber was added with medium (30% FBS) for 16 h incubation at 37 °C. Migrated cells were fixed with 4% paraformaldehyde and stained with 0.1% crystal violet. After that, cell graphs were taken by a microscope to analyze and calculate the migration rates.

### Human apoptosis antibody array analysis

LV-shCtrl and LV-shPSMC2-transfected high metastasis HCC cells (HCCLM3) were collected to extract total proteins and the proteins’ concentration was measured via a BCA Protein Assay Kit (Pierce, Logan, UT, USA). Human Apoptosis Antibody Array (Abcam, Cambridge, MA, USA) was adopted according to the manufacturer’s instructions, and spots on the array membrane were imaged by a camera.

### RNA sequencing

Total RNA samples from stable shPSMC2 expressing- SK-HEP-1 cells and control cells were separated using Trizol instructed by the manufacturer’s manuals. RNA quality was determined by Thermo NanoDrop 2000 (Waltham, MA, USA) and Agilent 2100 Bioanalyzer (Palo Alto, CA, USA). IVT was detected via 3′IVT Plus kit (Affymetrix, Santa Clara, CA, USA) according to the manufacturer’s instructions. GeneChip Hybridization Wash and Stain Kit was used and the effects were analyzed using GeneChip Scanner 3000 (Affymetrix, Santa Clara, CA, USA). Raw data’s quality was assessed and filtered with R studio. |Fold Change| > 2.0 and FDR < 0.05 (*P* values obtained via Benjamini–Hochberg analysis) were used for significant difference genes screening criteria. IPA was performed and |*Z* score| > 2 is considered statistically significant.

### Mice xenograft model

Four-week-old, specific pathogen-free female BALB/c nude mice were purchased from Shanghai SLAC Laboratory Animal Co., Ltd (Shanghai, China) and housed in an environmentally controlled condition (22 °C with a 12 h light/dark cycle). All 20 mice were randomly divided into two groups. Stably transfected shPSMC2 or shCtrl HCCLM3 (0.2 mL cell suspensions with 2 × 10^7^ cells) were subcutaneously injected into the right back of each mouse. The measurement of mice weight, length (L) and width (W) of each tumor was conducted two times per week. The volume of tumors was estimated using volume = 3.14/6 × L × W × W. At 27 days post cell injection, all mice were isoflurane gas anaesthetized to be taken in vivo fluorescence imaging by the Perkin Elmer IVIS Spectrum (Waltham, MA, USA). After mice were sacrificed, tumors were all removed for weighing and photographing. All studies on mice performed in our study were approved by Institutional Animal Care and Use Committee of the First Affiliated Hospital, Zhengzhou University.

### Statistical analysis

All assays were accomplished in triplicate. Data were presented as mean ± SD. Significant difference between different groups were estimated by the two-tailed Student’s *t* test or one-way ANOVA. The differences of PSMC2 expression patterns revealed in IHC were assessed using Sign test, Mann–Whitney *U* analysis, and Spearman rank correlation analysis. Statistical significance (*P* value) was obtained according to SPSS 22.0 (IBM, SPSS, Chicago, IL, USA), where *P* value < 0.05 manifested a statistically significant difference. GraphPad Prism 6.01 (GraphPad Software, La Jolla, CA, USA) was performed for graphing during the whole analysis.

## Supplementary information

Table S1

Table S2

Table S3

Supplementary figure legends

Figure S1

Figure S2

Figure S3

Figure S4

Figure S5

Figure S6

Figure S7
