# Supplementary material for: PSMC2/ITGA6 axis plays critical role in the development and progression of hepatocellular carcinoma
Source: Cell Death Discov. 2021 Aug 19;7:217. doi: 10.1038/s41420-021-00585-y (PMC8376978; doi:10.1038/s41420-021-00585-y)
Supplement: Supplementary file 1 — Table S1 [file 41420_2021_585_MOESM1_ESM.docx]

Table S1 Antibodies used in western blotting and IHC

| Primary antibodies | Dilution in WB | Source species | Company | Catalog No. |
| --- | --- | --- | --- | --- |
| PSMC2 | 1:1000 | Mouse | Santa Cruz | SC-166972 |
| GAPDH | 1:3000 | Rabbit | Bioworld | AP0063 |
| EGFR | 1:1000 | Rabbit | abcam | ab52894 |
| EGR1 | 1:500 | Rabbit | Bioss | bs-1076R |
| ITGA6 | 1:500 | Rabbit | Bioss | bs-2641R |
| Caveolin-1 | 1:500 | Rabbit | abcam | ab2910 |
| Akt | 1:1000 | Rabbit | CST | 4685 |
| p-Akt | 1:1000 | Rabbit | bioss | bs-5193r |
| CDK6 | 1:1000 | Rabbit | abcam | ab151247 |
| TLR4 | 1:1000 | Mouse | santa cruz | sc-293072 |
| PIK3CA | 1:1000 | Rabbit | abcam | ab40776 |
| Primary antibodies | Dilution in IHC | Source species | Company | Catalog No. |
| PSMC2 | 1:100 | Rabbit | Santa-Cruz | SC-166972 |
| ITGA6 | 1:200 | Rabbit | Bioss | bs-2641R |
|  |  |  |  |  |
|  |  |  |  |  |
|  |  |  |  |  |
| Secondary antibody | Dilution |  | Company | Catalog No. |
| HRP Goat Anti-Rabbit IgG (WB) | 1:3000 |  | Beyotime | A0208 |
| HRP Goat Anti-Mouse IgG (WB) | 1:3000 |  | Beyotime | A0216 |
| HRP Goat Anti-Rabbit IgG (IHC) | 1:200 |  | Abcam | Ab111909 |
