# Supplementary material for: PSMC2/ITGA6 axis plays critical role in the development and progression of hepatocellular carcinoma
Source: Cell Death Discov. 2021 Aug 19;7:217. doi: 10.1038/s41420-021-00585-y (PMC8376978; doi:10.1038/s41420-021-00585-y)
Supplement: Supplementary file 2 — Table S2 [file 41420_2021_585_MOESM2_ESM.docx]

Table S2 Primers used in qPCR

| Gene | Forward primer sequence (5’-3’) | Reverse primer sequence (5’-3’) |
| --- | --- | --- |
| GAPDH | TGACTTCAACAGCGACACCCA | CACCCTGTTGCTGTAGCCAAA |
| PSMC2 | CAGCACTCTGGGATTTGGCT | TTTCTATCCACGCCCACTCTC |
| CAV1 | TCTGGGGCATTTACTTCGC | GATGGAATAGACACGGCTGATG |
| PPP2R5E | TCATGGACACGCTATCTGATCT | TGCTCTGTCAAACAGCCTCTGC |
| CCNA2 | AGCCTGCGTTCACCATTCA | GGGCATCTTCACGCTCTATTTT |
| PRKACB | TGGATTGGTGGGCATTAGG | GAACTGAAGTGGGATGGGAAT |
| CCND3 | TACCTGGATCGCTACCTGTCTT | GGTCGGTGTAGATGCACAGTTTT |
| CDK5R1 | GAAGGCCACGCTGTTTGAGGAT | AGGCAGCACGGAGATGATGGAG |
| PTGS2 | CAAATCCTTGCTGTTCCCACC | TTTCTCCATAGAATCCTGTCCG |
| CUL3 | GCCTTGACAAATCAACGGAA | ACATGCAACCAAGGTCTTCTG |
| SMAD2 | TCCATCTTGCCATTCACGC | CCACTTTTCTTCCTGCCCATT |
| TGFBR1 | GTCATCACCTGGCCTTGGTC | GGTCCTCTTCATTTGGCACTC |
| EGFR | ATGAGGACATAACCAGCCACC | AGGCACGAGTAACAAGCTCAC |
| UBE2N | ATCCGCACAGTTCTGCTATCG | TATGGCTTGGGCTTCGTTG |
| EGR1 | CACCTGACCGCAGAGTCTTTT | TGGTTTGGCTGGGGTAACTG |
| EIF4E | AAAACAAACGGGGAGGACG | CAACAGCGCCACATACATCAT |
| FOS | CAGACTACGAGGCGTCATCC | TCTGCGGGTGAGTGGTAGTA |
| FOXO1 | ACCCAGCCCAAACTACCAA | ACTGACTCATACCTCCATAACTCG |
| IRS1 | GGTGGATGACTCTGTGGTGG | GGACGCTGATGGGGTTAGAG |
| ITGA6 | GCTCCCAGAGCCAATCACAG | CCGCCACATCATAGCCAAAT |
| MRAS | TGACAACCTCCCCACATACAA | TGGGGTCATAGTCAGGCACA |
| PPP2CA | GTTCCCCATGAGGGTCCAAT | TTGCCCAAAGGTGTAACCAG |
