# Supplementary material for: PSMC2/ITGA6 axis plays critical role in the development and progression of hepatocellular carcinoma
Source: Cell Death Discov. 2021 Aug 19;7:217. doi: 10.1038/s41420-021-00585-y (PMC8376978; doi:10.1038/s41420-021-00585-y)
Supplement: Supplementary file 3 — Table S3 [file 41420_2021_585_MOESM3_ESM.docx]

Table S3 Relationship between PSMC2 expression and tumor characteristics in patients with HCC analyzed by Pearson correlation analysis

| Tumor characteristics | index |  |
| --- | --- | --- |
| Gender | Pearson correlation | -0.218 |
|  | Significance (two tailed) | 0.023 |
|  | n | 108 |
| T [Infiltrate](D:/360%E5%AE%89%E5%85%A8%E6%B5%8F%E8%A7%88%E5%99%A8%E4%B8%8B%E8%BD%BD/Dict/8.4.0.0/resultui/html/index.html#/javascript:;) | Pearson correlation | 0.242 |
|  | Significance (two tailed) | 0.012 |
|  | n | 108 |
| Stage | Pearson correlation | 0.208 |
|  | Significance (two tailed) | 0.031 |
|  | n | 108 |
