# Supplementary material for: PSMC2/ITGA6 axis plays critical role in the development and progression of hepatocellular carcinoma
Source: Cell Death Discov. 2021 Aug 19;7:217. doi: 10.1038/s41420-021-00585-y (PMC8376978; doi:10.1038/s41420-021-00585-y)
Supplement: Supplementary file 4 — Supplementary figure legends [file 41420_2021_585_MOESM4_ESM.docx]

**Figure S1.** (A) The transfection efficiencies of shPSMC2 and shCtrl in BEL-7404 and SK-HEP-1 cells were evaluated through observing the fluorescence of GFP on lentivirus vector. (B) The knockdown efficiencies of 3 shRNA for silencing PSMC2 were evaluated by qPCR in SK-HEP-1 cells. Data was shown as mean ± SD. ***P* < 0.01

**Figure S2.** The expression of Caspase-3 and Caspase-7 in shCtrl and shPSMC2 BEL-7404 and SK-HEP-1 cells was detected by western blotting.

**Figure S3.** (A) The transfection efficiencies of shPSMC2 and shCtrl in HCCLM3 were assess by fluorescence imaging. (B) The knockdown of PSMC2 was confirmed by qPCR and western blotting, respectively. (C) MTT assay was performed to evaluate the effects of PSMC2 knockdown on HCCLM3 cell proliferation. (D) The effects of PSMC2 knockdown on HCCLM3 cell apoptosis were evaluated by flow cytometry. Data was shown as mean ± SD. ***P* < 0.01, ****P* < 0.001

**Figure S4.** (A) The volcano plot of gene expression profiling in SK-HEP-1 cells with or without PSMC2 knockdown. Green dots represented significantly downregulated DEGs; red dots represented significantly upregulated DEGs. (B) The enrichment of the DEGs in canonical signaling pathways was analyzed by IPA. (C) The enrichment of the DEGs in IPA disease and function was analyzed by IPA. (D) The schematic diagram of CDK5 signaling pathway. (E) The correlation of PSMC2 and ITGA6 expression was analyzed by the data collected from TCGA database.

**Figure S5.** (A) The data collected from GSE121248 showed the upregulation of ITGA6 in HCC tissues compared with normal ones. (B) The data collected from TCGA showed the significant correlation between ITGA6 high expression and poor prognosis of HCC patients.

**Figure S6.** (A) The transfection efficiencies of shCtrl, shITGA6, shPSMC2+shITGA6 in SK-HEP-1 cells were evaluated through observing the fluorescence of GFP on lentivirus vector. (B) The knockdown efficiencies of 3 shRNAs designed for ITGA6 knockdown were evaluated by qPCR. Data was shown as mean ± SD. **P* < 0.05

**Figure S7.** The expression of Caspase-3 and Caspase-7 in SK-HEP-1 cells with mere PSMC2 knockdown or ITGA6 knockdown, or simultaneous PSMC2 and ITGA6 knockdown was detected by western blotting.
