# Supplementary figures and images for: PSMC2/ITGA6 axis plays critical role in the development and progression of hepatocellular carcinoma
Source: Cell Death Discov. 2021 Aug 19;7:217. doi: 10.1038/s41420-021-00585-y (PMC8376978; doi:10.1038/s41420-021-00585-y)

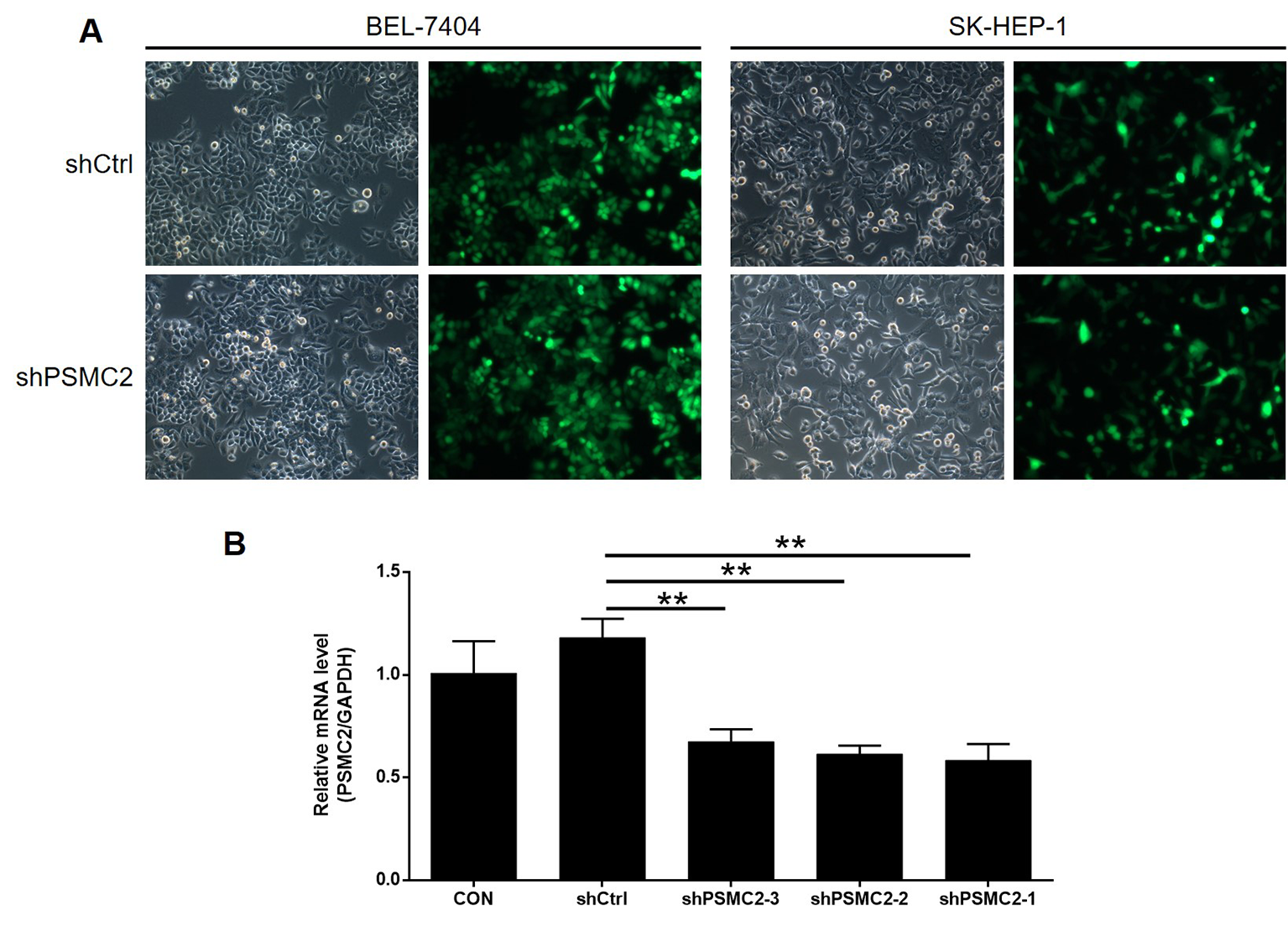

Supplement: Supplementary file 5 — Figure S1 [file 41420_2021_585_MOESM5_ESM.tif]

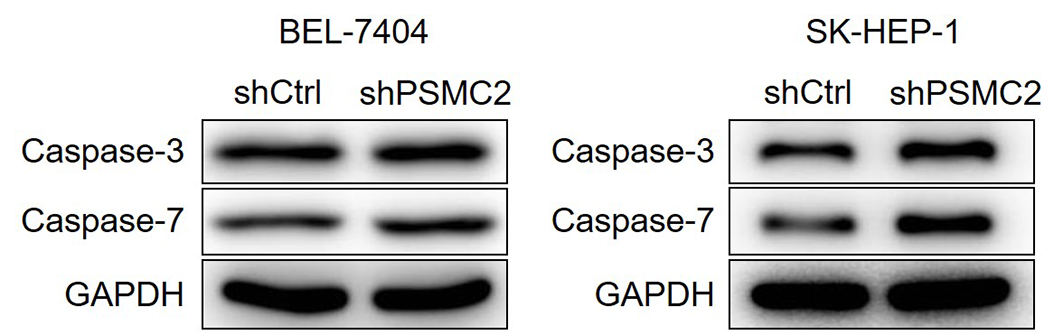

Supplement: Supplementary file 6 — Figure S2 [file 41420_2021_585_MOESM6_ESM.tif]

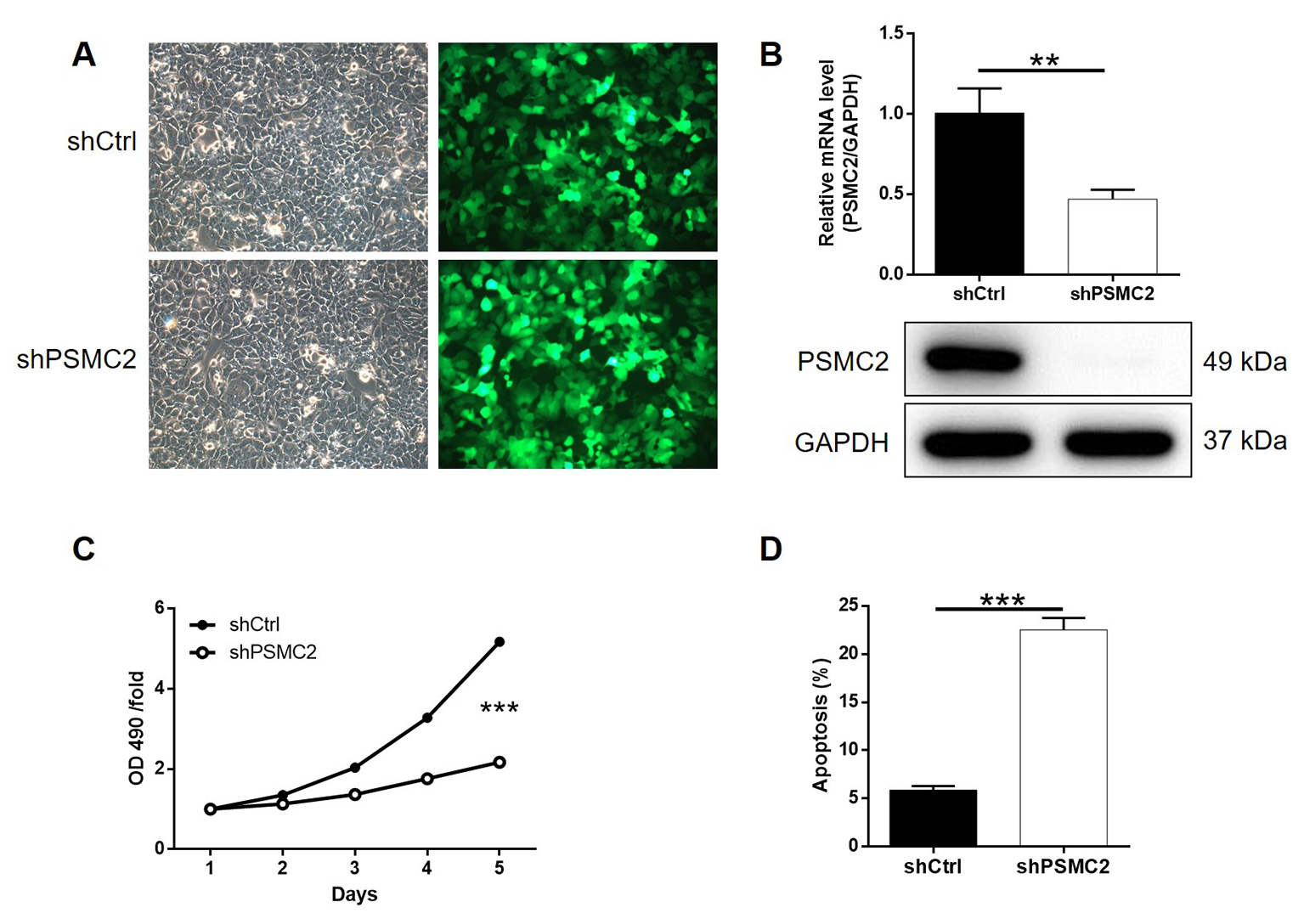

Supplement: Supplementary file 7 — Figure S3 [file 41420_2021_585_MOESM7_ESM.tif]

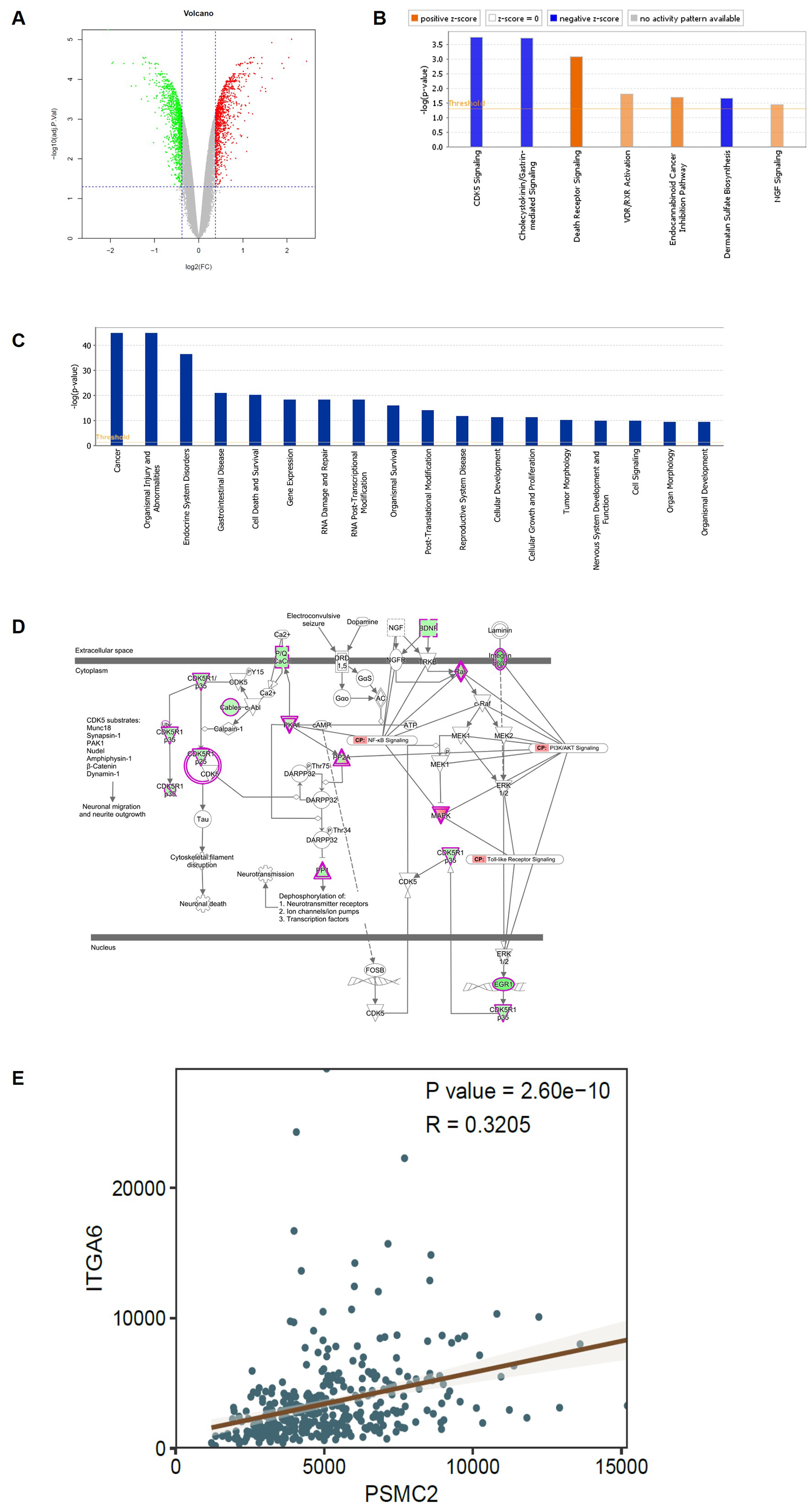

Supplement: Supplementary file 8 — Figure S4 [file 41420_2021_585_MOESM8_ESM.tif]

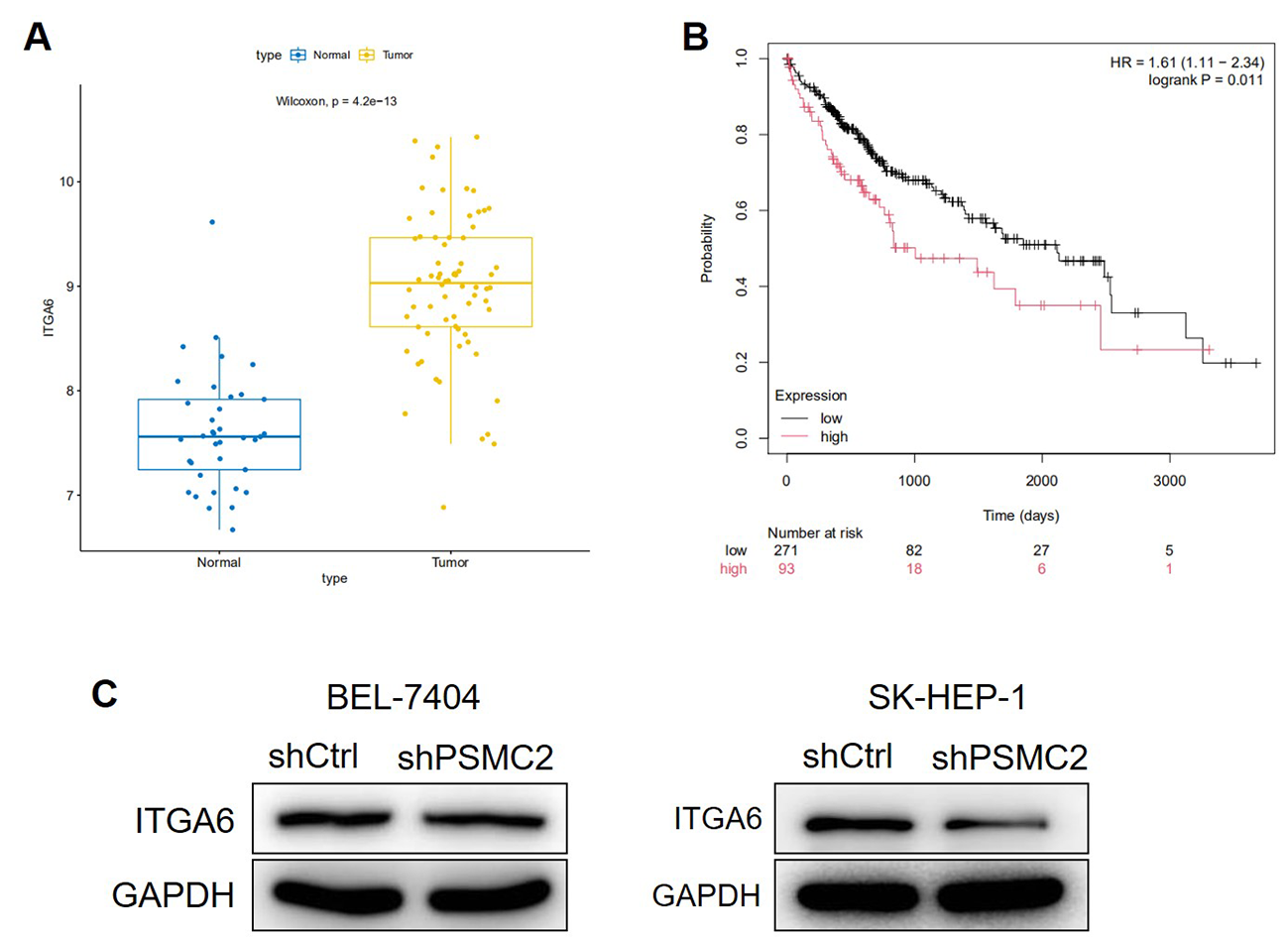

Supplement: Supplementary file 9 — Figure S5 [file 41420_2021_585_MOESM9_ESM.tif]

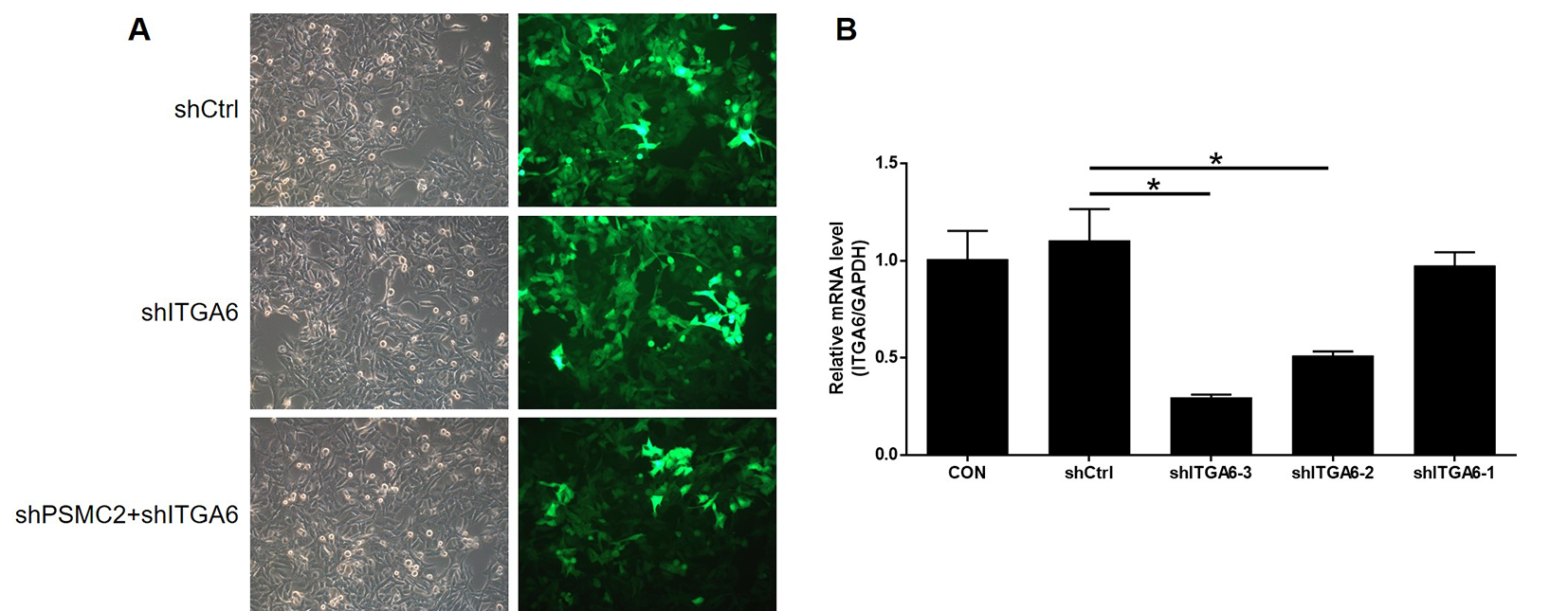

Supplement: Supplementary file 10 — Figure S6 [file 41420_2021_585_MOESM10_ESM.tif]

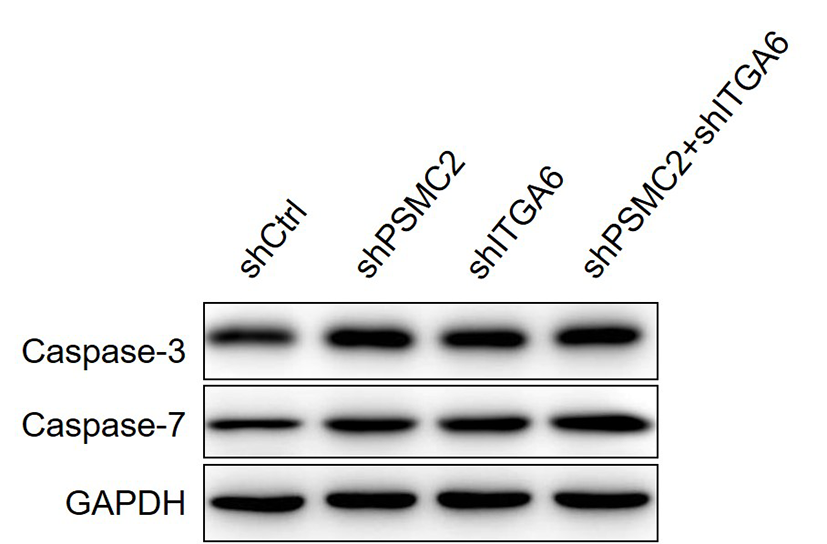

Supplement: Supplementary file 11 — Figure S7 [file 41420_2021_585_MOESM11_ESM.tif]
